# The mechanical properties of the spinal cord: a protocol for a systematic review of previous testing procedures and results

**DOI:** 10.1186/s13643-023-02417-9

**Published:** 2024-02-07

**Authors:** Megan Stanners, Marguerite O’Riordan, Laura Hartley, Eirini Theodosiou, Jean-Baptiste Souppez, Adrian Gardner

**Affiliations:** 1 Aston University, Aston Street, Birmingham, B4 7ET UK; 2 The Royal Orthopaedic Hospital NHS Foundation Trust, Bristol Road South, Northfield, Birmingham, B31 2AP UK

**Keywords:** Spinal cord, Spine, Model, Compression, Tension, Mechanical testing

## Abstract

**Background:**

Spinal cord compression is a pathology seen in routine clinical practice. However, there remain a number of unanswered questions around both the understanding of the pathogenesis and the best method of treatment of the condition. This is partly due to the issues of the real-life testing of the physical properties of the spinal cord, either through the use of cadaveric human specimens or through animal testing, both of which have methodological, as well as ethical, issues.

**Design and methods:**

This paper details a protocol for a systematic review of the literature on the mechanical properties of the spinal cord. We will conduct a literature search of a number of electronic databases, along with the grey literature, as a single-stage search. All literature will be screened for appropriate studies which will then be reviewed fully to extract relevant information on the methodology and mechanics of the reported testing along with the results. Two reviewers will separately screen and extract the data, with a comparison of results to ensure concordance. Conflicts will be resolved through discussion and independent arbitration as required. The methodological quality of the studies will be assessed within the ARRIVE guidelines using the CAMARADES framework and SYRCLE risk of bias tool. A narrative synthesis will be created with the appropriate tables to describe the demographics and findings of the included studies.

**Discussion:**

The systematic review described here will form the basis of an understanding of the current literature around the physical properties of the spinal cord. This will allow future work to develop a physical model of the spinal cord, which is translatable to patients for analysis and testing in a controlled and repeatable fashion. Such a model would be the basis for further clinical research to improve outcomes from this condition.

**Trial registration:**

Prospero registration number: CRD42022361933.

## Background

The spinal cord is a part of the central nervous system that connects the brain, housed inside the skull, to the rest of the body. Anatomically, the spinal cord exits the skull via the foramen magnum and passes inferiorly, through the vertebral foramen of each vertebral body, from C1 to L2 with nerve roots exiting the vertebral column at every level. Inferior to L2, the spinal cord becomes the cauda equina [1].

Compression of the spinal cord or nerve roots is a source of pain and disability for patients and this is often caused through degeneration of the intervertebral disc and posterior facetal articulations [2]. In some cases, this degeneration can also be associated with the development of malalignment of the individual vertebral bodies in relation to one another, such as is seen with spondylolisthesis [3]. Relieving neural compression through surgery is one of the functions of the spinal surgeon. Decompression of the spinal cord within the cervical spine has been described via a number of different philosophies and surgical approaches [4]. However, there continues to be a debate over the relative merits of the different methods employed [5–7], and questions such as whether an anterior or posterior approach (surgery through the front or back of the neck) should be performed for the best outcomes in relation to the health and long term function of the spinal cord, still divide the surgical community.

What is missing from the literature is knowledge that quantifies what happens to the spinal cord during compression and the subsequent surgical decompression in humans. Previous literature reports on a variety of animal models [8–13] to provide evidence as to the effect on the spinal cord of applying both compression and tension forces. To move away from experimenting on animals and to be able to obtain data that is applicable to humans, a physical model of the spinal cord and vertebral column is required. Using this model, the different scenarios that occur clinically in the cervical spine (compression of the spinal cord with or without kyphosis and/or spondylolisthesis) could be simulated. Furthermore, the effects on the spinal cord of the different surgical procedures that are performed for cervical spinal cord compression (anterior decompression, posterior decompression, spinal fusion, cervical disc replacement, and spinal realignment procedures) could also be simulated.

This review aims to examine and understand how the material properties of the spinal cord have previously been reported in the literature. The work is part of data gathering, which will inform future work on the creation of a reproducible in vitro spine-analog, to enable quantitative experiments on the spinal cord for various clinical scenarios.

## Design and methods

This protocol has been designed by experts in the field of biological engineering and spinal surgery. The protocol will be devised in line with the Preferred Reporting Items for Systematic Review and Meta-Analysis Protocol (PRISMA-S) methodology [14, 15]. It has also been registered in the International Prospective Register of Systematic Reviews (PROSPERO ID–CRD42022361933). The method will involve conducting a single-step search of electronic databases. This search will identify those papers that describe a model of spinal cord function. All literature in the final review will be assessed within the ARRIVE guidelines [16] using the CAMARADES framework [17] and Syrcle Risk of Bias tool [18]. The identified papers will be critically appraised as described below.

### Eligibility criteria

**Fig. 1:**
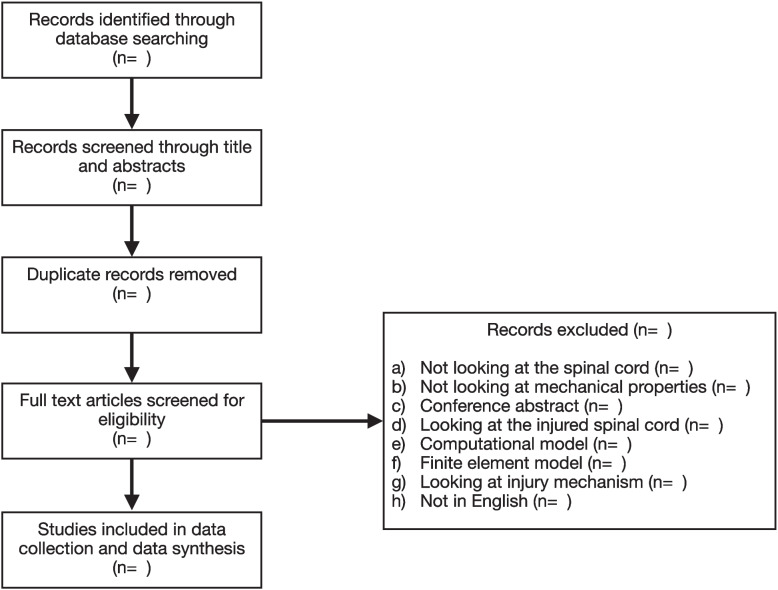
The projected study flowchart

The population to be included in this review are all types of studies that investigate the mechanical properties of the spinal cord. Both human and animal models will be accepted. The intervention of interest is the mechanical testing of the spinal cord in any fashion. There will be no comparator group. The outcomes of interest are the mechanical properties of the spinal cord. These may include measures of uniaxial tension and compression, which will be used to determine levels of resistance to deformation and which will give an indication of the stiffness, toughness, strength, hardness, and brittleness of the spine. Studies that do not describe the results of a mechanical measure will not be included. There will be no limits placed on study design or geographical location for the included papers, but only studies published in English will be included (Fig. 1).

### Information sources

The search strategy will involve systematic searches of the electronic databases AMED, BNI, CINAHL, EMBASE, EMCARE, MEDLINE, PsychINFO, and PubMed from the date of first entry to the date of search, via the Healthcare Databases Advanced Search (HDAS) system [19]. There will be no limits set on the time period that the studies were published. Grey literature will also be searched [20, 21].

### Search strategy

The search will be performed via blocks of text and appropriate syntax using the search terms: (mechanical*)AND(properties*)AND(spinal cord*). No filters will be added. All search results will be reviewed by the first author (MS). The second author (MOR) will independently review 25% of the total studies to ensure concordance between the two reviewers. MS and MOR will pilot the data collection by comparing their results from their independent review after five reviewed papers. Studies will be reviewed based on both the title and abstract of the paper and then classified as either relevant, irrelevant, or unsure based on the defined eligibility criteria. Any studies considered irrelevant based on title and abstract will be excluded. Any disagreements will be settled via an independent arbitrator (AG). Studies deemed relevant by the independent reviewers will proceed to critical appraisal, where the full text will be reviewed and re-categorized into either relevant or irrelevant. In the circumstance where the reviewers cannot agree on the relevance of the study, independent arbitration will occur (AG).

### Data management

Papers deemed relevant for further full-text review will be downloaded from HDAS [19] as a Word document. Included in this document will be: the study number, the title of the study, the study author(s), the lead institution, the publication date and type, the PubMed ID of the study, the abstract of the study, and the database where the study was found.

### Data extraction

From each paper, information regarding demographics, species, sample storage, sample preparation, and mechanical testing parameters, will be extracted and put into a number of data collection tables (see Table 1 for proposed data to be extracted to the data tables). Inquiries will be made of the corresponding author if the required data is incomplete or unclear. This will be done by MS who will independently assess the eligible papers and complete the data collection tables with the data items. MOR will randomly assess 25% of the studies and independently complete the data collection tables. Comparisons between the two reviewers will be made to ensure coherency of results. Any unresolvable differences between the reviewers will be resolved by an independent arbitrator (AG).

**Table 1:** Data items to be extracted

Selected studies details		Sample preparation and storage		Sample mechanical testing	
Item	Data type	Item	Data type	Item	Data type
Country	Nominal	Storage method	Nominal	Test type	Nominal
Animal	Nominal	Storage temperature	Interval	Pre-conditioning	Interval
Age and stage	Interval	Storage time post-death	Interval	Number of samples per test	Interval
Weight	Interval	Thawing method	Nominal	Sample dimensions	Interval
Number of animals	Interval	Preparation prior to testing	Nominal	Pre-load	Interval
Live, dead, or euthanized	Nominal			Test rate	Interval
Anatomical location	Nominal			Sampling frequency	Interval
				Test temperature	Interval
				Test humidity	Interval
				ISO standard used	Categorical

### Critical appraisal of studies

The selected articles will be critically appraised within the framework of the Animal Research: Reporting of In Vivo Experiments (ARRIVE) guidelines [16] using the CAMARADES framework [17] and Syrcle Risk of Bias tool [18]. In the same fashion, as detailed above in data extraction, this critical appraisal of studies will be carried out by MS and MOR with unresolvable differences resolved by an independent arbitrator (AG).

### Data analysis

All data on the mechanical properties of the spinal cord from the reviewed literature will be presented. It is highly likely that most of the results will be from a number of different animal models. All results that show sufficient homogeneity will then be pooled. To allow quantitative pooling of data, the source data will need to be comparable in terms of the mechanical properties being assessed. From the initial assessment of the literature, the authors anticipate that it will not be possible to pool the data so a narrative synthesis of the literature will be produced following the Systematic Review without Meta-Analysis (SWiM) guidance [22]. However, if it is appropriate, subgroup analysis will be subsequently performed by individual mechanical properties. Data analysis will be performed by all authors.

## Discussion

Several solutions are currently employed in clinical practice to address compression of the spinal cord and spinal nerve roots. These solutions differ in both the surgical approach taken to the spine and the technique of decompression. Despite the ongoing debate as to the relative benefits of the various techniques within the medical literature, there is an overall agreement that a full decompression of compressed structures is required [5–7]. The decision on what surgery is performed will depend on a number of factors, including the previous experience and training of the surgeon, the characteristics of the pathology on MRI and CT scans, and patient factors that might rule out at least one of the potential surgical options. What does not form part of that list is quantifiable data as to what the effects of the pathology, and subsequent surgery, are at the level of the spinal cord, and this review is the first step in addressing this knowledge gap. Knowing how the spinal cord behaves as a material will add to the information available to treating clinicians, on how to best manage a particular clinical scenario, in addition to the other components of decision-making commented on above.

The aim of the overarching research programme, of which this proposed review is part, is to allow the creation of a physical in vitro, fully articulated model of the vertebral column and spinal cord. This model would go some way to solving the ethical issues related to experiments on humans (which would be highly illegal) or animals (which have moral sensitivities [23] with the subsequent issues around the generalisability of the results from animals into humans [24]). Via the model, both the pathology and the surgery can be simulated with quantifiable measures to demonstrate the outcomes on the spinal cord. Given the advances in 3D imaging and printing, the model could be tailored to individuals, and therefore allow patient-specific management to be undertaken and the results known prior to surgery.

Whilst this proposed model would replicate the mechanical properties, the physiological function of the spinal cord is to transfer information via neurons within the matter of the cord [25]. Prolonged compression of the spinal cord results in progressive failure of those neurons [26] with paralysis as the end result, through loss of sensation or motor power below the level of cord compression [27]. It is envisaged that the ultimate goal of all of this work would be the creation of a model that, in addition to reproducing the mechanical properties of the spinal cord, has complex physiological functions that could be measured during simulated pathology and surgery.

### Protocol amendments

Any amendment that is made to the protocol whilst conducting the systematic review will be detailed clearly in the published article and will be updated in PROSPERO.

### Ethics and dissemination statement

This review does not require ethical approval to proceed. Patients and the general public will not be consulted in the construction of this review. Consent to participate is unnecessary for the purpose of this review. The findings of this systematic review will be published in the peer-reviewed literature and presented at national and international conferences.

## Data Availability

The datasets used and/or analyzed in this review are available from the subsequent author on reasonable request.

## Abbreviations

3D: Three dimensional
HDAS: Healthcare Databases Advanced Search
CT: Computed tomography
MRI: Magnetic resonance imaging
PRISMA: Preferred Reporting Items for Systematic Review and Meta-Analysis Protocol
SWiM: Systematic Review without Meta-Analysis
